# Steroid responsiveness of peripheral blood T cells derived from steroid sensitive, steroid dependent, and steroid resistant asthmatics, and induction of steroid resistance by costimulatory signal

**DOI:** 10.1186/2045-7022-3-S1-P5

**Published:** 2013-05-03

**Authors:** Akio Mori, Akemi Abe, Satoshi Kouyama, Miyako Yamaguchi, Yo Iijima, Chihiro Mitsui, Chiyako Oshikata, Hidenori Tanimoto, Kiyoshi Sekiya, Takahiro Tsuburai, Masami Taniguchi, Mamoru Ohtomo, Yuji Maeda, Maki Hasegawa, Kazuo Akiyama, Takayuki Ohtomo, Osamu Kaminuma

**Affiliations:** 1 National Hospital Organization, Sagamihara National Hospital, Clinical Research Center, Japan; 2 Tokyo University of Pharmacy and Life Science, Department of Pharmacotherapeutics, Japan; 3 The Tokyo Metropolitan Institute of Medical Science, Department of Allergy and Immunology, Japan

## Background

Severe asthmatics are characterized by low responsiveness to inhaled corticosteroid (ICS) compared to mild asthmatics. Steroid resistance has been ascribed to various cell types including T cells, mononuclear cells, bronchial smooth muscle cells, etc.

## Method

Peripheral blood mononuclear cells (PBMC) obtained from mild (steroid sensitive, SS), steroid dependent (SD), and steroid resistant (SR) asthmatics were stimulated with either mitogens or allergens. Effects of glucocorticoids (GCs) on the proliferation and cytokine synthesis were analyzed. Der f 2-specific Th clones were established from PBMC by the limiting dilution.

## Results

IL-5 production by PBMC of SS asthmatics was significantly reduced after ICS administration, but that of SD asthma remained high. IC_50_ values of dexamethasone suppression on cytokine synthesis and proliferation was not statistically different among SS, SD, or SR asthmatics. Addition of CD28 signal made proliferation of anti-CD3-stimulated Th clones steroid-resistant. The induction of steroid resistance was dependent on IL-2 receptor signal and PI-3 kinase activity.

## Conclusion

Besides T cell intrinsic mechanisms, steroid responsiveness of T cells seems to be determined by the microenvironment, costimulatory signals and cytokines. Costimulatory signal might be involved in the induction of steroid resistance in T cells of SD asthma. The notion is consistent with our recent finding that administration of CTLA4-Ig made SR asthma model SS.

